# Dr. Sidney Allan Fox

**Published:** 1891-01

**Authors:** 


Dr. Sidney Allan Fox died of pneumonia at his home in Brooklyn, December 11, 1890, aged thirty-three years. He was a specialist in diseases of the nose, throat and lungs, in which department of medicine he was fast making a name. He was a delegate to the Berlin Medical Congress from the Medical Society of the State of New York. He married the daughter of Hon. William J. Coombs, and she is now herself ill with pneumonia.

